# How can peer teaching influence the development of medical students? a descriptive, longitudinal interview study

**DOI:** 10.1186/s12909-023-04801-4

**Published:** 2023-11-13

**Authors:** Marijke Avonts, Katrien Bombeke, Nele R. Michels, Olivier M. Vanderveken, Benedicte Y. De Winter

**Affiliations:** 1 Skills Lab, Faculty of Medicine and Health Sciences, University of Antwerp, Antwerp, Belgium; 2 Department of Family Medicine and Population Health, Faculty of Medicine and Health Sciences, University of Antwerp, Antwerp, Belgium; 3 Otolaryngology and Head and Neck Surgery, Antwerp University Hospital, Antwerp, Belgium; 4 Laboratory of Experimental Medicine and Pediatrics, University of Antwerp, Antwerp, Belgium

**Keywords:** Peer teaching, CanMEDS, Self-efficacy, Skills training, Interviewing study, Role model, Maturity, Medical student, Personal growth, Professional growth

## Abstract

**Background:**

Peer-assisted learning (PAL) – where students take up a teaching role at an early stage of their training—is widely used in medical curricula. Many qualitative studies have investigated the perceptions and benefits of PAL, but no studies have longitudinally explored how peer teachers experienced their development. This could allow for a better understanding of PAL. In this study, we explored the perceived impact of being a peer teacher on the development of personal and professional competencies as a medical student.

**Methods:**

We longitudinally conducted semi-structured interviews with peer teachers, during their 2-year teaching period in the skills lab at the University of Antwerp and applied descriptive thematic analysis.

**Results:**

In total we gathered 47 interviews in 13 peer teachers (9 female, 4 male,. 1–7 interviews each). Peer teachers reported an increase in self-confidence, which gradually transformed into self-efficacy in clinical and teaching skills., Participants told us to be inspired by the previous generation of peer teachers. Their motivation shifted from personal benefits to benefiting others while becoming a role model themselves. The peer teachers illustrated how they developed maturity by integrating different CanMEDS roles. They grew in reflection, changed/transformed an initial mark-driven study drive into more patient-centered ambitions, and started developing a personal style.

**Conclusions:**

Our study suggests that being a peer teacher leads to more self-efficacy, in clinical and teaching skills, to become a role model with as motivation to benefit others and to grow towards a good doctor maturity. Although the task is to teach peers, this opportunity nurtures the practice and integration of various CanMEDS roles, not only that of scholar but also communicator, collaborator and leader, thereby positively influencing their personal and professional development and their identity as a doctor (professional role).

**Supplementary Information:**

The online version contains supplementary material available at 10.1186/s12909-023-04801-4.

## Background

While peer teaching has been implemented in undergraduate education since the early 1990s, the popularity of peer teaching activities within medical education increased since 2000 [1]. Peer teaching is now widespread and a part of medical curricula in the five continents [2].

Nearly all authors [3] refer to the definition of peer teaching made by Topping: “People of similar social groupings who are not professional teachers helping each other to learn and learning themselves by teaching” [4]. Peer assisted learning (PAL) is a commonly used umbrella term and encompasses teaching methods where students learn from students.

Teaching is an essential skill for future doctors as teaching students and patients is an important part of a doctor’s activities [5, 6]. Despite the perceived importance of residents’ teaching roles, they may not receive adequate formal training in teaching skills [5, 7]. Therefore, Cohen et al. proposes to implement peer teaching at the undergraduate level to prepare these future residents for their teaching role [8].

Two recent reviews described how PAL is currently implemented in medical schools: most PAL programs teach rather practical items (skills, anatomy) [9]; are elective [8] for students and are relatively short in duration (less than 3 months) [8, 9].

As most PAL programs are elective, it is interesting to determine why students apply. A first reason is to be able to review all the educational material [10–12] and to improve their own skills [10, 12, 13]. Peer teachers enjoy teaching [11], want to help other students [10, 12] and hope to network with other students and faculty [12, 13]. Finally, they also state that it fosters their professional development [12] and career opportunities [13].

The CanMEDS Framework is a competency model designed by the Royal College of Physicians and Surgeons of Canada. It focusses on the abilities needed by all physicians to meet het health care needs of the patients, communities, and societies they serve [14]. This framework is organized around seven ‘roles’ that physicians need to meet: Medical Expert (ME), Communicator (CR), Collaborator (CL), Health Advocate (HA), Leader (LR), Scholar (S), and Professional (P). The key competencies of the professional role are developing a professional attitude, reflection on their own behaviour and recognizing their own limitations[14–16]. This is in accordance to the definition of professionalism of Goldie [17] and in accordance with how students develop a professional identity, i.e. ways of being and relating in professional contexts [18].

What is the impact of peer teaching? PAL enables medical students to assume teaching roles, leading to improved learning outcomes [19], as teaching cultivates an intrinsic motivation to study their courses [3, 20]. Peer teachers report an improvement of their teaching skills and feel prepared and motivated for future teaching roles [8, 9]. Moreover, peer teachers also highlight developmental advantages such as increased self-confidence and the ability to embrace uncertainty [3].

While the studies mentioned have examined the impact of peer teaching at one or two moments in time, there is currently a gap in the literature as no studies have longitudinally tracked the development of peer teachers during PAL [9, 21].

Only Yeung et al. followed medical students during an extracurricular peer teaching program lasting a full academic year. The peer teacher’s confidence in teaching and answering questions was significantly increased and they improved their own learning. The longitudinal format was very well appreciated in preparation of their future teaching roles [22].

As the development of competencies and learning evolves over time [6, 8], a more longitudinal follow-up could explore if peer teachers undergo an evolution through the program or change their perceptions. Competencies are not directly measurable, but refer to a quality [23].This enables a better understanding of how peer teachers develop personally and professionally.

Our previous, retrospective cohort study concluded that high-performing students self-select as peer teachers and suggested that being a peer teacher supports the development of certain CanMEDS roles (medical expert, collaborator, scholar and professional) [24].

In this qualitative study, we explore the perceived impact of being a peer teacher on the development of personal and professional competencies as a medical student.

## Methods

### Context

**Fig. 1:**
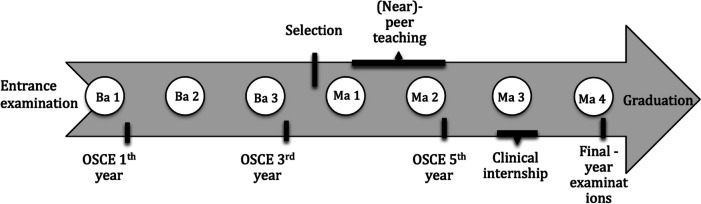
Timeline of the 7-year medical curriculum at University of Antwerp. Ba: Bachelor; Ma: Master; OSCE: Objective Structured Clinical Examination

The curriculum of the medical school at the University of Antwerp has been adapted to the CanMEDS framework [25] During medical school students at the University of Antwerp can apply for a voluntary peer teaching program in the Skills Lab during their 4^th^ and/or 5^th^ year (equaling the 1^st^ and 2^nd^ Master Year, Fig. 1). This is 1 or 2 years before the students start a full year of clinical internships and after obtaining their Bachelor’s degree (Fig. 1).

We cite the concept of our PAL program from our previous study [24]: “The selection of the students was based on a completed application form, in addition to a curriculum vitae and a cover letter explaining their motivation to become a peer-teacher. When the peer teachers were selected (by 2 faculty members from the Skills Lab), they would choose 1 or 2 topics out of a list of all OSCE stations to teach: heart and lung, abdomen, basic life support (BLS) and first aid, suturing, intramuscular (IM) injection and drawing blood, gynecology, musculoskeletal examination, neurology, eye examination and ear, nose, throat (ENT) examination, taping, and examination of a neonate. Subsequently, the selected students received training of approximately 2 h per teaching topic from an experienced staff member. During this training, they practiced the necessary clinical, physical, and technical skills and received advice on how to teach and provide feedback (didactic skills).

In this peer teaching program students teach the different physical examination skills to their fellow students (3^rd^ to 5^th^ year) and coach them during additional practice sessions. This provides voluntary training sessions for students, in addition to the official skills training. It also supports students in mastering the necessary skills and provides an ideal preparation for the OSCE (Objective Structured Clinical Examination) that takes place at the end of both the 3^rd^ and 5^th^ years of medical school. Students need to succeed at these high stakes OSCEs before they can enter a Master’s program or a fulltime clinical internship year.

During the peer skills sessions, 5 to 8 peer teachers present for up to 30 to 50 students. Each peer teacher provides 3 to 5 sessions a year. For each topic a coordinator is appointed for the group of peer teachers. This student is responsible for all the communication between the peer-teachers and with the faculty concerning the organization and practical aspects of the training sessions. These coordinators are supervised by the faculty staff member(s) from the Skills Lab.” [24].^1^

### Study participants

In 2014–2015 there were 32 peer teachers, 17 from Master 1 and 15 from Master 2. All peer teachers from Master 1 were invited to participate. We selected these students so we could track them for a period of 4 semesters (2 years). We also sent them an e-mail containing the study information before their first peer teaching session. At that session, a researcher was present to answer any questions that the students had. Ethical approval was received and informed consent was obtained from all participants.

### Study design

We aimed to explore how being a peer teacher influences the development of personal and professional competencies among medical students using a longitudinal qualitative interview study. Semi-structured interviews were used to gather data on peer teachers’ experiences over time.

The peer teachers were tracked during their 2-year career as a peer teacher, starting from Master 1 (just after their selection) until the end of Master 2, covering 2 to 13 sessions of peer teaching. This variability is due to the number of topics a peer teacher teaches and the durations (1 year or 2 years) they are active in the program.

After each peer teaching session, we interviewed the student as soon as possible to gain insight on their experiences during the past session (Table 1). For the interview guide, we developed questions that we assumed would stimulate the students to spontaneously talk about their ‘lived’ experiences. The interviewer was well briefed about the aim of the study and used that information to go deeper on subjects brought by the students without hinting.

**Table 1:** Topics guiding the interviews

Can you describe what exactly you have been doing during these practice sessions?
How did it go? How was it for you?
Did things go the way you expected?
What do you particularly remember from this practice session?
What do you particularly remember about yourself after this session?
Did you notice anything in particular about yourself during this practice session?
Did you notice or learn anything about yourself that could still be useful to you as a doctor?

We alternated short face-to-face interviews with interviews by mail. The latter consists of an initial mailing followed by a response by the interviewer with specific questions, to collect all the relevant information. Both interview types were performed by 2 experienced interviewers (LS and KB). We mentioned clearly that their quotes would not have any impact on their career and the interviews were anonymized for further analyses. The face-to-face interviews were recorded and transcribed verbatim for analyses.

### Analysies

Leaning more towards a ‘realist’ research perspective (‘small-q’) [26] perspective we used descriptive thematic analysis for generating themes within our qualitative data. Realism broadly assumes that there are things ‘out there’ in the world that have a real, objective existence [27], although they cannot apprehended directly because they are processed through our brain, language, culture and methods. In this way, we explored both the peer teachers experiences as shared in the interviews, and underlying causal powers [27] inferred by rigorous analysis, generating codes and themes in different steps.

**Fig. 2:**
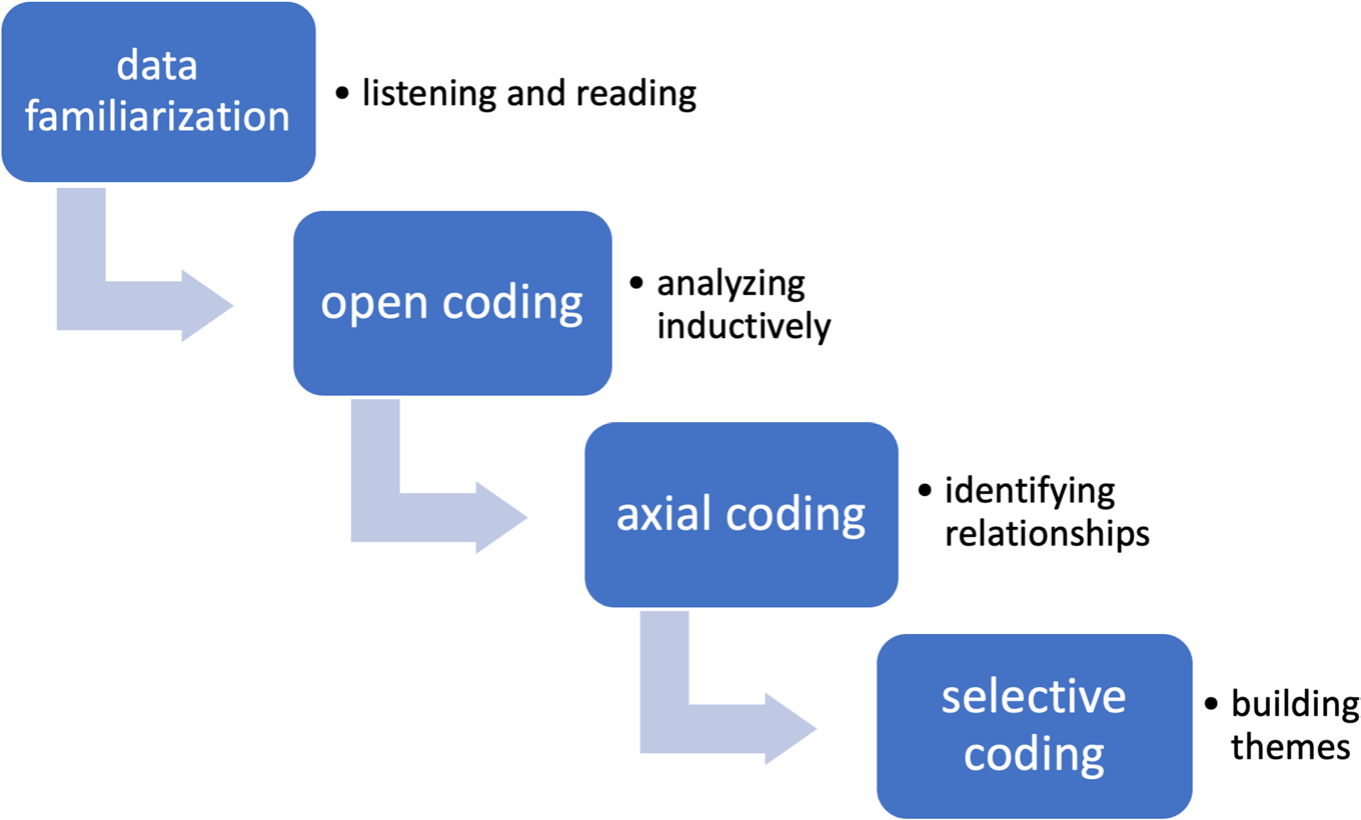
Different steps in our thematic analyses

In the first step (Fig. 2), data familiarization was achieved by listening to all the interviews and reading through the transcripts. All interviews were coded by 2 researchers (MA, KB) independently. We used open coding; this means we started analyzing inductively without any pre-set codes. After every 5 interviews there was a consultation between MA and KB to compare codes, discuss them, and modify them until a consensus was reached. All codes were assembled into a codebook (Table 2, the complete codebook in supplement).

**Table 2:** Summary of the codebook

Aggregated codes	Individual codes
Motivation for peer teaching	
Hidden curriculum	
Affected by peer teaching	Sense of responsibility Self-confidence of peer teachers Emotions of peer teachers
Learning	Peer teacher learning Development as a peer teacher
Impact of peer teaching	Return as peer teachers OSCE
Teaching	
Interaction with	Faculty staff Peer teachers Medical students
Different practice sessions	
Experience as coordinator (leading)	
Plans for the future	
Practical issues	
Introspection	Dealing with failings Reflection – peer teacher as a person
Focus on delivering quality	
Extracurricular activities	
Connection with patients	

Subsequently, MA performed the analyses and discussed periodically with KB. All discrepancies were discussed until agreement was achieved.

**Fig. 3:**
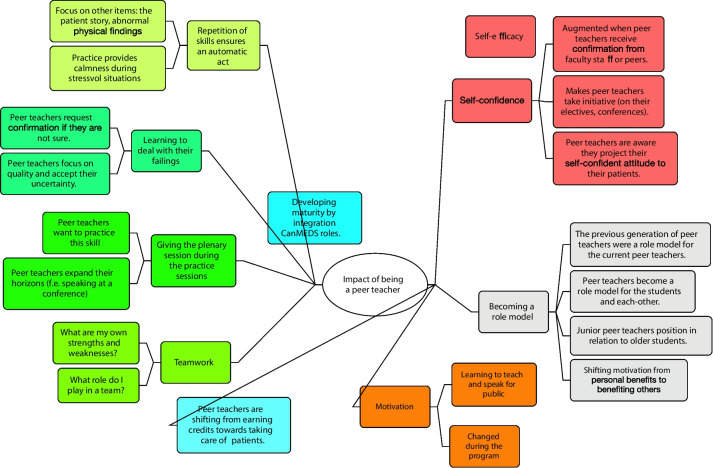
Mind map of relationships identified during axial coding

After initial open coding, we identified relationships (axial coding) between the individual codes by inductive reasoning (MA and KB). An example: we created the relationship ‘teamwork’ after combining all the codes where peer teachers reflected about themselves as being part of a group and noticing all the interactions between the different peer teachers. Figure 3 provides an overview of all relationships created during axial coding presented as a mind map.

Next, we chose a ‘core concept’ for analysis (selective coding) and built ‘themes’ to better understand what students learned from being a peer teacher (MA and KB). The different themes were revised toward a general thematic framework. The themes were chosen because of their recurrence within the interviews, their relevance for our research questions, and/or their innovativeness.

The CanMEDS roles informed our inductive analysis as ‘sensitizing concepts’ [28], i.e. we were aware of it, but it did not structurally guide our analysis as a framework. In a final selective coding step, we reviewed all the codes related to the themes at different time points for each student. This allowed us to better understand the experienced impact of PAL/peer teaching over time. During the process we had several meetings to promote critical reflection with all investigators. This researcher triangulation was performed to enhance trustworthiness. We performed the analysis with the aid of NVivo 12.

After analysis, we contacted all peer teachers (*n* = 13) to return the results and check for accuracy and resonance with their experiences, known as member checking. Six of them reacted and agreed with most of the headlines of this study.

## Results

Thirteen peer teachers (9 female, 4 male) agreed to participate. Three of them stopped being a peer teacher after 1 year. The other 10 peer teachers were tracked for 2 years. In total we gathered 47 interviews (34 face-to-face interviews and 13 mailings) (Table 3).

**Table 3:** Summary of peer teachers

	**# sessions (Y1/Y2)**	**# years**	**# interviews/mailings**	**sex**	**Topic**	**Motivation**
Peer teacher 1	6 (1/5)	2	4 (3/1)	F	intramuscular (IM) injection and drawing blood, BLS and first aid	e, g
Peer teacher 2	3 (3/0)	1	2 (2/0)	F	Eye examination & Ear Nose Throat (ENT)	a
Peer teacher 3	9 (5/4)	2	6 (3/3)	F	Suturing, heart and lung, taping, gynaecology	a
Peer teacher 4	5 (1/4)	2	3 (2/1)	F	Musculoskeletal examination, eye examination and &ENT, examination of a neonate	a, g
Peer teacher 5	9 (4/5)	2	5 (4/1)	F	Suturing, IM injection and drawing blood, taping	a
Peer teacher 6	6 (1/5)	2	2 (2/0)	M	Musculoskeletal examination, abdomen	a,c
Peer teacher 7	9 (2/7)	2	3 (2/1)	F	BLS and first aid, suturing, abdomen	
Peer teacher 8	7 (2/5)	2	3 (2/1)	M	BLS and first aid, IM injection and drawing blood	f
Peer teacher 9	9 (3/6)	2	6 (4/2)	M	Heart and lung, BLS and first aid	b, d
Peer teacher 10	12 (5/7)	2	7 (5/2)	M	Suturing, gynaecology, abdomen	b, d
Peer teacher 11	6 (3/3)	2	3 (2/1)	F	Suturing, abdomen	c
Peer teacher 12	2 (2/0)	1	2 (2/0)	F	Eye examination and ENT	e
Peer teacher 13	3 (3/0)	1	1 (1/0)	F	Eye examination and ENT	
Total			47 (34/13)			

Table 2 illustrates the most relevant codes generated through our analyses addressing a variety of aspects concerning PAL. During analysis we built ‘themes’ to understand how peer teachers experienced their PAL period. These themes are presented in a mind map in Fig. 3. The 4 themes identified were 1) change in motivation 2) self-efficacy in clinical and teaching skills, 3) becoming a role model and 4) developing maturity by integrating the CanMEDS roles [14].

In the following section, we indicated the occurrence of different CanMEDS roles using the previously mentioned abbreviations (background), f.e. results based on interview data referring to the role of the Medical Expert is indicated (ME).

### Changing motivation from personal benefit to helping others

There were different reasons for each peer teacher to enroll in the program (Tables 3 and 4). Most peer teachers applied for the program to develop certain skills (as speaking in front of a group or learning to teach). Some students were inspired by the previous peer teachers and wanted to offer the students the same chances of practicing skills they had experienced (S).

**Table 4:** Reasons for students to become a peer teacher

a) Learning to teach and speak in public
b) To practice their skills
c) Enjoying the act of teaching
d) To help others
e) They had good experiences with peer teaching while they were students
f) To learn from other peer teachers
g) It offers career opportunities

During the interviews several peer teachers indicated that their motivation had changed over time. One peer teacher admitted having started the program for his own development, but he felt so satisfied by the positive responses of the students that teaching others became his new motive (S). Another peer teacher enjoyed the way his teaching skills developed and learned that every doctor needs these skills in their professional life (P). It enhanced his motivation. Illustrating quotes are displayed in Table 5.

**Table 5:** Quotes illustrating the change in motivation

PT 3 – Interview 2 (Master 1, Semester 2)	*” I will do the plenary explanation next session otherwise it did not made sense to register as a peer teacher. I have to cross that border. It’s difficult, but I need to.”*
PT 10 – Interview 5 (Master 2, Semester 4)	*“In one of the previous interviews, I mentioned that I do the peer teaching mainly for myself. By teaching others, I can learn new things myself. When I noticed that the other students learnt new things, this gave me a big sense of accomplishment, and this is another reason why I decided to become a peer teacher.”*
PT 8—Interview 2 (Master 2, semester 3)	*“This year I pay more attention to how I can learn from my colleagues to give a clear explanation and hope to improve this skill. I think that whatever doctor I will be, passing through information will always be part of my job* *That was definitely not my initial motivation to become peer teacher.”*

### More self-efficacy in clinical and teaching skills

Peer teachers developed confidence in their clinical and teaching skills. This was built through the affirmation that they received from staff members, students, and fellow peer teachers. Peer teachers also learned through the questions that students asked them, especially when they were challenged to really think about their subject matter. If the student did not understand it, this made them realize that there can be different views and they learned to use a different perspective in their teaching (S). Some peer teachers indicated that they maybe didn’t improve their skills, but above all they felt more confident in their skills through the program. They also demonstrated their self-confidence to (simulated) patients during consultation practice lessons. This is an important step in building the doctor-patient relationship, even more so during 'embarrassing' examinations, such as a gynecological exam (CR).

The peer teachers reported to take more initiative because of their increased self-confidence in clinical skills during electives (e.g., requesting to suture wounds on patients). Furthermore, peer teachers learned to become calmer during stressful situations. Here, they mainly referred to the stressful OSCE exam. An Illustrating quote is displayed in Table 6.

**Table 6:** Quote illustrating self-efficacy in examination skills

PT 10 Interview 6 (Master 2 – Semester 2)	*“Last week, we had a consultation training with a simulated patient where I had a gynecological problem to deal with. Last times, I already reported that being a peer teacher makes me more confident. I experienced it again. Although the examination was not perfect because you don’t know what kind of problem it will be in advance, I managed to do it quite well and I was confident because of I am a peer teacher for these skills.“*

### Becoming a role model for their peers

Peer teachers were becoming a role model for their students and each other as they developed their clinical and teaching skills and inspired others. Peer teachers felt responsible for effectively teaching skills. In doing so, they encountered their own shortcomings and learned to deal with them (P). Unanimously, they indicated that they had no problems consulting a colleague or a present teacher (CL). They learned that it is normal to occasionally feel insecure and to not know something (P). Even as a future doctor, this can occur. After all, you cannot know everything, they answered firmly. An Illustrating quote is displayed in Table 7.

**Table 7:** Quote illustrating role modeling

Peer teacher 2 – Interview 2 (Master 1, Semester 2)	*“Yes, a doctor can't know everything. I think that it’s perfectly fine to acknowledge when you don’t know something by saying ‘I don’t know’. It actually benefits your patients when you can admit your limitations and seek assistance from others.”*

### Developing maturity

We defined mature students as students who have more life experiences outside the standard curriculum. During the interviews the peer teachers provided insight into how they developed maturity by integrating the different CanMEDS roles.

The practice sessions always started with a short plenary session. Peer teachers stepped out of their comfort zone (P) by speaking in front of a group (S) together with their peers (CL) which stimulated them to reflect on their communications skills (CR) afterwards. Doing this simple act, 4 different CanMEDS roles were addressed.

Peer teachers rehearsed the skills of their topic very regularly. As a result, they developed a routine (ME). Some even called it an 'automaticity'. This approach gave them a sense of clarity, allowing them to fully focus on the patient's history (CR) and enhancing their clinical diagnostic reasoning skills (ME).

The peer teachers worked in small groups of 3 to 6 peer teachers per topic and were all responsible for the content of the practice session. This taught them how to function in a team (CL, P). They reflected on their own strengths and weaknesses as team members and thought about how they aimed to function in a team the next year during their internship (CL).

Based on the peer teachers’ experiences PAL seemed to stimulate the process of 'learning to obtain good grades in exams’, to 'learning to be a good doctor'. The tipping point was situated at the transition from Bachelor to Master. They realized that the purpose of learning for an examination was not to perform it on a simulation patient, but rather to understand the underlying cause of the patient’s symptoms (ME, HA). They occasionally felt a bit uncomfortable when students from lower academic years were primarily concerned with acquiring skills for upcoming exams rather than prioritizing their development as future doctors.

As a result, they derived enjoyment when they were asked to assist in training professionals, f.e. a workshop suturing for sailors. They then experienced how the questions asked were very focused on what was happening in practice.

By working on the same material each time, the peer teachers expressed noticing how they made the material their own and developed even their own style. Illustrating quotes are displayed in Table 8.

**Table 8:** Quotes illustrating developing maturity

Peer teacher 7 – Interview 3 (Master 2, Semester 4)	*… “If something abnormal arises or if there’s a procedure you’re unsure about, it doesn’t disrupt your entire flow. For example, if you suddenly notice that someone has a scar, you can take a moment to think logically about how to proceed. Should you avoid that area or proceed with palpitation? This way you maintain your continuity of thought. (…) It’s similar to when you’re cooking an egg. You know that first you’ll put the water on the stove, and then get an egg and put it in the water. You don’t have to think about the steps.”* Peer teacher 7 (interview 3: Master 2, semester 4)
Peer teacher 5 – Interview 4 (Master 2, Semester 4)	*"The audience consisted mostly of sailors. The questions were very practically oriented … about Steri-Strips, skin glue (when to use each, …) and the most common question was 'suppose I'm x hours from shore, is it best to suture myself or do I wait until I get to the doctor?' or 'within how much time should it be sutured?' … So, the questions were more practical and broader in scope compared to regular practice sessions.”*

### Longitudinal perspective

Although the analysis is based on the data of all participants, we now present 4 cases to better illustrate the influence of being a peer teacher on medical students’ personal and professional development over time. Each quote used in this section is contextualized within the specific timeframe of the peer teachers' academic journey, denoting their respective semesters in Master 1 (semester 1 and 2) and Master 2 (semester 3 and 4).

#### Peer teacher 3: “It is sometimes difficult for me to speak in front of a large group.”

This peer teacher not only learned to present to a group, but also experienced that she is allowed to make mistakes. She has learned to step out of her comfort zone and to find her place in a team (P, CL).

By doing this regularly, they developed confidence. Even when unexpected situations arise, such as when fellow peer teachers were absent, and they had to deal with the class on their own (P). Illustrating quotes are displayed in Table 9.

**Table 9:** Quotes from peer teacher 3

Interview 1 (Master 1, semester 2)	“*At present, I do feel prepared, but initially, I struggled with giving explanations to the entire group. If I would have to explain something to the entire class, then I would prefer to be more thoroughly prepared. Whenever I find myself in front of a group, tasked with explaining something, there’s always a tendency to overlook certain details.”* “*I also realized that when you receive questions, that it prompts you to think and also learn something yourself. If you’re not completely certain about something, then you can seek guidance from others or a professor that is present, which enables you to learn from the experience.”*
Interview 3 (Master 1, semester 2)	*"During the gynaecology practice session, I was the only peer teacher present at the start of the session. … It was also the first time that I gave the explanation at the start of the session, which was quite exhilarating for me as I always find speaking in front of a large group to be an exciting experience.”*
Interview 6 (Master 2, Semester 4)	(About the day she was alone for the practice session gynaecology.) *“Later on, when everything went well, I felt a sense of relief. It made me realize that I might not have done the same thing if I was with others, and it was a valuable lesson for me. I learned that I can confidently explain things in front of a large group without feeling too nervous.”* *“Having the mindset that 'you don't have to know everything' is also beneficial (…). Initially, as a peer teacher I was overly cautious about not saying anything incorrect (…). This mindset made me more nervous than necessary.”* *"I think that when I'm in a team that I should assert myself more. It’s crucial not to completely fade into the background and to ensure that my voice is heard.”*

#### Peer teacher 12: “It's about humans.”

This student made it very clear how she slowly grew into her role as a future doctor and how important it was for her that the previous peer teachers acted as role models.

The previous generation of peer teachers acted as mentors for the current peer teachers, providing an example of the desired characteristics of an effective peer teacher. Illustrating quotes are displayed in Table 10.

**Table 10:** Quotes from peer teacher 12

Interview 1 (Master 1, Semester 1)	*"I'm not an experienced peer teacher yet, so I haven’t fully developed my own style. However, when I attended practice sessions (…), I learned important skills from those who helped me (…). I remember thinking, that’s how I want to do it too.”*
Interview 2 (Master 1, Semester 2)	*"I think it’s important to highlight this more. It’s not just about exam performance, e.g., ‘'if you do that on your exam, you will fail'. But it’s also about the potential risks such as infections and needlestick injuries, both during exams and in real-life situations. (…) We need to remember that it ultimately concerns the well-being of people"* *“As a peer teacher now, I want to teach students that it doesn't have to be perfect. It’s about knowing the right order and understanding how to perform examinations, so that you have confidence in what you are doing”* *"Through experience, you become more familiar with the tools you use and develop your own techniques. For example, I have short fingers, so I learned to adjust my grip on a nasal speculum by holding it more towards the base. This way you learn some tips and tricks that enhance your skills, while also learning what specific details you need to observe”*

#### Peer teacher 10: “I find it amazing how much confidence that students have in us.”

This student learned to reflect on his own learning and experiences to take more initiative.

Unlike many other peer teachers, he revealed himself to be a rather nonchalant student who planned everything at the last minute.

As the sessions progressed and he further developed into his role as a peer teacher, he reflected on his preparation. He recognized that he was no longer solely responsible for his own teaching, but that he now had the responsibility of teaching others.

He literally said how his motivation shifted from personal benefits towards benefiting others and how amazed he was by the confidence students had in the peer teachers. He was becoming a role model himself. Illustrating quotes are displayed in Table 11.

**Table 11:** Quotes from peer teacher 10

Interview 2 Master1, Semester 2	*"I tend to be someone who often completes tasks at the last minute and relies on the belief that if I encounter difficulties, I can find information just in time to figure out how to do it (…). I don’t typically prepare in advance.”*
Interview 4 Master 2, Semester 3	*“With every question the students ask, you end up learning something yourself, don’t you?* *Even if you don't know and you need to ask someone, then you should absolutely do so (…). At the end, you may not be able to list the specific things you learned, but you gained self-confidence."* *"Perhaps for me that's just a way to boost my confidence, I don't know. Because maybe I can't do better. (…) The feeling maybe, but that's important too."*
Interview 5 Master 2, Semester 4	*“After the explanation, during the students' practice session, I noticed that we had forgotten to mention some helpful tips and practical information. Although this wasn’t a major problem, I learned from this experience the importance of being more systematic and structured during the explanation and presentation. This way we can ensure that nothing essential is overlooked.”* *“In one of the previous interviews, I mentioned that I do the peer teaching mainly for myself. By teaching others, I can learn new things myself. When I noticed that the other students learnt new things, this gave me a big sense of accomplishment, and this is another reason why I decided to become a peer teacher.”*
Interview 6 Master 2, Semester 4	*"In gynaecology, I think that it’s important to come across as being confident, considering that it can be an embarrassing examination for patients (…). When a doctor displays confidence, it greatly helps the patient to feel more comfortable and relaxed."*

#### Peer teacher 8: "Teaching isn't really for me."

This peer teacher saw in his peers a clear role model who helped him grow into a better teacher. Throughout the interviews his teaching skills and his self-confidence as a teacher grew.

For most peer teachers, it was a conscious decision to sign up for PAL. In the case of Peer teacher 8, we saw a different story. He responded to a call launched because peer teachers were still needed for a particular topic. Given his hobby as an ambulance driver, he knew he had enough expertise, but did not see himself as a 'teacher' at all. Hoping to grow and develop, he became a peer teacher. Throughout the interviews he provided feedback on how he observed fellow peer teachers during practice sessions (their teaching style, how they improve students) and he followed these examples to further develop his own teaching skills (P). Illustrating quotes are displayed in Table 12.

**Table 12:** Quotes from peer teacher 8

Interview 1 (Master 1, Semester 2)	*"At the beginning I was not really planning on becoming a peer teacher, but at the start of this year I received an email that peer teachers were still needed for first aid and CPR. So I sent in my application at the last-minute."*
Interview 2 (Master 2, Semester 3)	*"I actually think that teaching is a role that maybe is not really for me, (…) I make things a bit too complex, I think. (…)"* *"I saw some very well-structured explanations (from other peer teachers) (…), especially last year. So, when I had the opportunity to give an explanation at the start of a practice session, I learned a lot from it. (…) I focused on having a clear structure, repeating things, and summarizing everything at the end. This helps to ensure that the students remember the information."* *"Yes, I don't think that I will ever really have the talent or the intrinsic capacity to be a teacher. However, through my experience with information transfer now (…), I feel that I have developed some competence in that area now.”*

## Discussion

Our longitudinal approach with multiple interviews provided a unique impression of how these peer teachers evolved over a 2-year time period. To the best of our knowledge, this is the first study where peer teachers were frequently interviewed during the whole process of peer teaching. This enabled us to explore how peer teachers changed their initial motivation form rather personal motives to helping others, how they developed self-efficacy in their clinical and teaching skills, how they became a role model and finally how they became mature students, developing a professional identity by integrating different CanMEDS roles.

Peer teaching involved many different steps (f.e. preparation, teaching and training, giving feedback, working together, reflection, tackle problems or unexpected events). Every one of these assignments challenged peer teachers to develop themselves in different roles as medical expert, teacher, communicator, collaborator, leader, professional. This integration of various CanMEDS roles positively influenced their professional development. Developing self-confidence and learning that this made them calmer in more difficult circumstances refers to self-efficacy toward their clinical examination skills on simulated and real patients. This concept from Bandura's social learning theory refers to a person's expectation about their ability to realize a (desired) behaviour [29]. It refers to beliefs about one's ability to perform a specific behaviour in specific situations. Peer teachers expressed the development of self-efficacy among peer teachers in their clinical and teaching skills by training, coaching and receiving the necessary trust from the faculty. Artino [30] reported that experiences, along with observing others, have an important impact on evaluating one’s own self-efficacy. Peer teaching, particularly in clinical skills, encompasses both of these elements. The peer teachers described how their experiences influenced their views of their own self-efficacy. Bandura [29] further emphasized that effective performance in a specific domain relies on both possessing the necessary skills and having the belief in one’s own ability to execute them effectively. Students need both ‘the skill and the will’ to function successfully.

Bandura also stated that people learn from one another via observation, imitation, and modelling [29]. Role modelling remains very important for medical students as a method of transmitting medical professionalism [31]. Good role models have a high standard of clinical competence, a good teaching ability and act as a successful team leader [31]. These are all qualities peer teachers are challenged to explore and develop during PAL. PAL is an ideal medium for role modeling as this process occurs mostly informal and unplanned [27, 32]. Our findings are consistent with other studies about this topic [13, 21, 33, 34]. Burgess et al. revealed the important function of clinical (faculty) tutors as role models [35] where our data suggests this can also be the case for peer teachers. Being a role model, peer teachers felt a responsibility to deliver quality teaching and training. A positive role model can play an important role in developing a professional identity [18, 35]. This identity is influenced more by the informal and hidden curriculum than by formal teaching experiences [18]. Peer teachers stated how they transformed from being assessment driven to an understanding that the skills are needed to be a good doctor. They experienced to be insecure as they cannot know everything. Peer teachers reported to reflect on their behaviour as student, learner and team member. Also reflection can be an important dynamic of personality change [18]. Peer teaching seemed to influence several aspects involved in developing professionalism.

The main motivation to enroll in the peer teaching program was the ability to learn to speak in public and teach other students. This is consistent with a similar study [13]. Engels et al. investigated how different reward categories were perceived by former peer teachers and concluded that ‘supporting others’ was appreciated the most [36]. As Kusurkar et al. stated, motivation is expected to be dynamic [37] and that is exactly what peer teachers reported in our longitudinal follow-up. During the program their motivation shifted from rather personal benefit towards helping others. These motives are excellent examples of how peer teachers are intrinsically motivated which is the desired type of motivation in students [38]. Another, more recent theory, the self-determination theory (SDT), relates to role theory and can explain why intrinsic motivation may benefit from being a teacher. SDT claims that intrinsic motivation is caused by three features: competence, autonomy, and relatedness to significant others [39]. Playing the role of a teacher may very well serve to create feelings in these three domains [40].

A medical career has been described as a series of transitions [41]. The longitudinal follow-up of these peer teachers gave insight in some important transitions they made from having no teaching experience to develop basic teaching skills needed as a doctor; from learning for grades to acting in the importance of the patient and from being afraid to stand in front of a group to have enough confidence to do this in the future. The transition from medical school to internship can be challenging and is a source of stress and anxiety for medical students [42]. A systematic review by Surmon et al. [43] revealed that students expressed their concerns over perceived deficiencies in knowledge and/or skills and that they are unsure about how to behave and act in a team. Surmon et al. also stated that maturity could impact preparedness for the internship as students with prior life experiences will adjust to the clinical environment more easily [41, 43]. Developing maturity may be due to a previous education, activities in their spare time but also extra-curricular activities like peer teaching.

The longitudinal experiences of peer teachers illustrated how they slowly transformed from students to junior doctors. Peer teachers felt more at ease in contact with (simulation) patients because of the routine that they had developed, which positively influenced their doctor-patient relationship. As peer teachers learned to embrace insecurity and became team members, they might be better prepared for the clinical workplace. Given the critical importance of the transition from classroom to workplace, this is an interesting topic for further investigation.

We also need to emphasize the limitations and strengths of our study. The transferability was limited as we only interviewed peer teachers at one institution. Our design provided their perceptions, opinions, and evolutions but we did not observe these students during peer teaching. Combined with the interviews, this could have given us a better understanding and a direct view on their development. Most interviews were face-to-face, but to be able to reach every peer teacher after each session we also included mailing. As mailings are less interactive than face-to-face interviews some more nuanced information may have been lost. The use of a qualitative approach and the provided privacy (LS did not have any teaching and/or assessing role towards the interviewed students, the interviews were recorded in a private room and anonymized for analysis) enabled potentially sensitive issues to be discussed in depth.

Future research questions regarding PAL of course remain:

- What impact does PAL have after peer teachers graduate? A follow-up after medical school could provide unique information.
- In the idea that all students should be able to gain educational skills in their basic medical curriculum, PAL could be made mandatory. If so, will PAL still have the same impact?

## Conclusion

Our study investigated the unexplored area of peer teachers’ perspectives during a longer clinical skills peer teaching project. In this longitudinal interviewing study peer teachers revealed how their initial motivation changed, how they developed self-efficacy in their clinical and teaching skills, how they became a role model and finally how they became mature students with a professional identity by integrating different CanMEDS roles. Although peer teachers’ main task is to teach, this opportunity allowed the practice, development and integration of other key professional roles. This contributes to their professional identity as a future physician.

### Practice points

- Every medical student should have the opportunity to be a peer teacher as this may provide the ability to become a role model, to develop maturity and to develop patient-centered skills.
- Peer teachers develop self-efficacy in clinical and teaching skills. Self-efficacy is created by training, coaching and receiving the necessary trust from faculty, but it is important that faculty remain available.

### Supplementary Information

**Additional file 1.**

## Data Availability

The datasets used during the current study are available from the corresponding author on reasonable request.

## Footnotes

1 Starting from 2012–2013 the University of Antwerp introduced a 6-year medical curriculum. The peer teachers in this study are the last generation of the previous 7-year curriculum.
