# Supplementary material for: How can peer teaching influence the development of medical students? a descriptive, longitudinal interview study
Source: BMC Med Educ. 2023 Nov 13;23:861. doi: 10.1186/s12909-023-04801-4 (PMC10644508; doi:10.1186/s12909-023-04801-4)
Supplement: Supplementary file 1 — Additional file 1. [file 12909_2023_4801_MOESM1_ESM.docx]

Codebook interviews peer teachers

| Name | Description | Files | References |
| --- | --- | --- | --- |
| Focus on delivering quality | Peer teachers demonstrate how to deliver quality by looking for the correct information during the practice session. | 19 | 28 |
| Appreciation of the practice session | Collection of answers on the question 'How did it go? How was it for you? | 32 | 39 |
| Teaching | Collection of skills linked to teaching. | 9 | 13 |
| Using their own experience as a peer teacher |  | 7 | 8 |
| Giving feedback |  | 9 | 15 |
| Teaching interactive |  | 2 | 2 |
| Teaching as a future doctor |  | 13 | 15 |
| Observing skills of students |  | 17 | 20 |
| Developing routine |  | 10 | 15 |
| Speaking in front |  | 26 | 38 |
| Answering questions - giving an explanation |  | 20 | 32 |
| Asking questions themselves |  | 5 | 5 |
| Motivation for peer teaching | Collection of items linked to the motivation of becoming a peer teacher. | 21 | 31 |
| Choice of teaching topic |  | 21 | 28 |
| Impact | Statements indicating the effect of being a peer teacher. | 0 | 0 |
| As peer teacher |  | 24 | 40 |
| On their own OSCE exam |  | 10 | 13 |
| Experience as a coordinator (leading) | Some peer teachers were coordinators of their group. What did they learn from this position? | 14 | 16 |
| Extracurricular activities | Overview of activities that peer teachers take up outside regular courses. | 13 | 17 |
| Guiding anatomy dissections |  | 2 | 3 |
| Feelings | Collection of statements related to the feelings experienced by peer teachers. | 0 | 0 |
| Receiving confirmation |  | 4 | 5 |
| Emotions of peer teacher |  | 1 | 1 |
| 'no' experience as a peer teacher |  | 19 | 26 |
| Teaching is fun |  | 16 | 19 |
| Being able to help people |  | 16 | 18 |
| Being nervous |  | 21 | 31 |
| Practice session is fun |  | 15 | 20 |
| Practice session is exhausting |  | 4 | 4 |
| Getting satisfaction |  | 11 | 14 |
| Feeling useful |  | 4 | 5 |
| Responsibility |  | 11 | 12 |
| Self-confidence peer teachers |  | 22 | 34 |
| Hidden curriculum | Overview of items related to the hidden curriculum during the practice session. | 20 | 36 |
| Interaction | Overview statements regarding the interaction between a peer teacher and another person. | 0 | 0 |
| Interaction wit faculty staff |  | 13 | 19 |
| Position to faculty staff |  | 2 | 4 |
| Interaction with students |  | 7 | 9 |
| Feedback from students |  | 9 | 10 |
| Position peer teacher vs group of students |  | 18 | 26 |
| It is easier to ask fellow students (instead of faculty staff) |  | 5 | 5 |
| Perception of peer teacher by student |  | 9 | 13 |
| Being amazed of the confidence students have in peer teachers |  | 4 | 4 |
| Teaching students something really useful |  | 9 | 12 |
| Students ask plenty of questions |  | 5 | 6 |
| Interaction with peer teachers |  | 10 | 12 |
| Consultation with other studocs |  | 16 | 19 |
| Making appointments |  | 10 | 11 |
| Position of peer teachers among themselves |  | 16 | 22 |
| Becoming a role model |  | 0 | 0 |
| Introspection | Quotes where peer teachers reflect on their own functioning. | 0 | 0 |
| Dealing with their own shortcomings |  | 18 | 33 |
| Reflection – peer teachers as a person |  | 25 | 50 |
| Learning | Collection of items where peer teachers indicate learning. | 0 | 0 |
| Learning path |  | 15 | 16 |
| Learning as a peer teacher |  | 0 | 0 |
| Development as a peer teacher |  | 20 | 35 |
| How have I learned |  | 22 | 41 |
| What have I learned |  | 20 | 27 |
| Personal interest of peer teacher |  | 1 | 1 |
| Preparation makes me more confident |  | 5 | 6 |
| Connection with patients | Overview of moments where peer teacher make a link to patients, f.e. (simulation) patients, internship | 1 | 2 |
| Patient-centered focus |  | 8 | 11 |
| Communication with patients |  | 6 | 6 |
| Effect of a confident physician on a patient |  | 7 | 8 |
| Practical items |  | 0 | 0 |
| Stressful curriculum |  | 3 | 3 |
| Organization of the practice sessions |  | 27 | 46 |
| Selection process peer teacher |  | 11 | 13 |
| Students attending the practice session |  | 3 | 5 |
| Preparation of students |  | 4 | 5 |
| Preparing practice session as a peer teacher |  | 19 | 23 |
| Confirmation of practice session came too late |  | 7 | 8 |
| Refreshing topic practice session |  | 16 | 19 |
| Training session as preparation for the practice sessions |  | 19 | 26 |
| Feeling prepared |  | 20 | 30 |
| What did I do during the practice sessions |  | 27 | 41 |
| Plans for the future | Collection of statements where peer teachers look to their own future. | 15 | 19 |
| Different practice sessions | Experience of students during practice session of different topics. | 8 | 10 |
